# A case of planar-type GIST of the sigmoid colon showing diverticular structure with perforation

**DOI:** 10.1186/s12957-020-01906-8

**Published:** 2020-06-11

**Authors:** Yuka Shintaku, Yuya Asano, Takahiro Watanabe, Takako Kihara, Eri Ishikawa, Yuan Jiayin, Neinei Kimura, Koji Kinoshita, Seiichi Hirota

**Affiliations:** 1 Department of Pathology, Hyogo College of Medicine, 1-1 Mukogawa-cho, Nishinomiya, Hyogo 663-8501 Japan; 2 Department of Pathology, Chibune Hospital, 3-4-39 Fuku-machi, Nishi-yodogawaku, Osaka, Osaka 555-0034 Japan; 3 Department of Surgery, Nomura-Kaihin Hospital, 2-1-41 Sumaura-dori, Suma-ku, Kobe, Hyogo 654-0055 Japan

**Keywords:** Gastrointestinal stromal tumor, GIST, Planar-type, Sigmoid colon, Diverticulum, Perforation, C-*kit* gene mutation

## Abstract

**Background:**

Gastrointestinal stromal tumors (GISTs) generally form well-defined mass lesions. However, some cases of the flatly distributed and muscularis propria-replacing GISTs have been reported so far. We experienced an additional case of planar-type GIST of the sigmoid colon accompanied by a diverticulum with perforation.

**Case presentation:**

A 68-year-old Japanese male with sudden onset of abdominal pain was clinically diagnosed with gastrointestinal perforation, and an emergency abdominal operation was performed. A diverticulum with rupture was found in the sigmoid colon, but no apparent tumor was observed. Histological examination revealed bland spindle cells flatly proliferating and diffusely replacing the muscularis propria at the diverticular structure. The spindle cells were positive for KIT, DOG1, and CD34. Mutational analysis of the c-*kit* gene revealed that the lesion had a heterozygous deletion of 2 amino acids at codons 557 and 558 of exon 11. The mutation was not observed in the normal mucosa of the surrounding tissue.

**Conclusion:**

We diagnosed this case as an unusual planar-type GIST. Some similar cases have been reported in the sigmoid colon and other sites. We discuss the mechanism of development of the planar-type GISTs associated with the diverticulum.

## Background

Gastrointestinal stromal tumors (GISTs) are the most common primary mesenchymal neoplasms of the gastrointestinal (GI) tract. GISTs are currently thought to be derived from the interstitial cells of Cajal (ICCs), the GI pacemaker cells [1]. Most GISTs are KIT-positive and have c-*kit* gene mutations [1]. These KIT signaling-driven neoplasms commonly originate in the stomach (60–70%) and small bowel including the duodenum (20–30%) and rectum (5–10%), but rarely occur in other GI tracts, especially in the colon. Approximately 10% of GISTs have platelet-derived growth factor receptor alpha (PDGFRA) gene mutations and most of them develop in the stomach.

GISTs generally form well-defined mass lesions. However, some cases of the flatly distributed and muscularis propria-replacing GISTs have been reported so far [2–7]. Here we present an additional case of planar-type GIST of the sigmoid colon accompanied by a diverticulum with perforation. We performed a literature review and discussed the mechanism of planar-type GIST formation.

## Case presentation

**Fig. 1:**
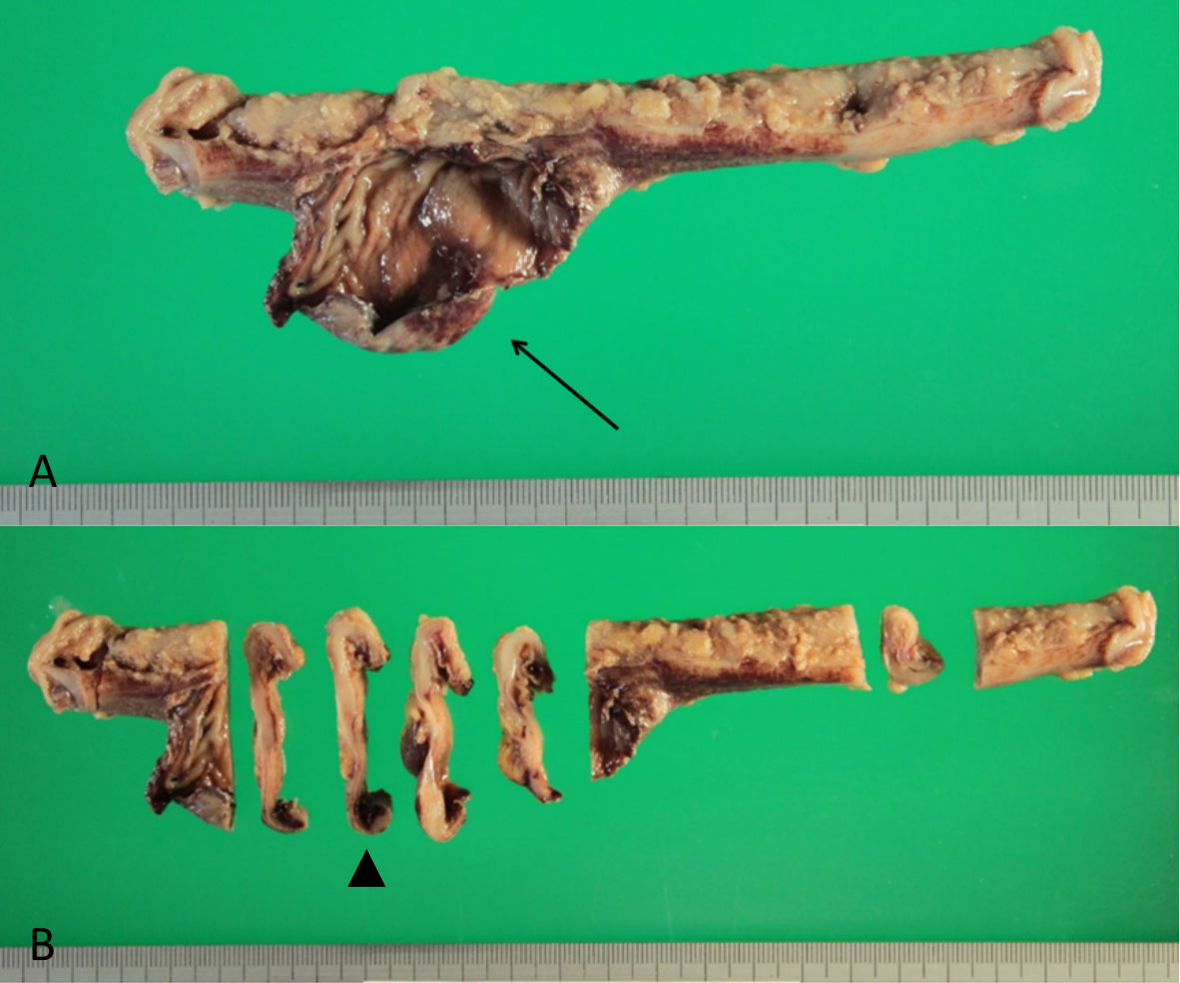
Macroscopic finding of the resected sigmoid colon. **a** A diverticulum with perforation (arrow) was observed. **b** The cut sections of **a** were shown. The histological specimens in Figs. 2 and 3 were made from the section indicated by the arrowhead

A 68-year-old Japanese male with sudden onset of abdominal pain was transferred to Nomura-Kaihin Hospital. He did not have any special past medical history and had never received colonoscopy. Contrast-enhanced computed tomography revealed a locally dilated sigmoid colon with fecaloma and perforation. Findings of feculent peritonitis (stage IV by Hinchey Classification) and free air in the peritoneal cavity were also detected, but no apparent tumor was pointed out. He was clinically diagnosed with perforation of the sigmoid colon, and an emergency operation was performed. During surgery, a diverticulum with wide frontage and rupture was found in the sigmoid colon, but no apparent tumor was observed. Fecaloma was present near the rupture site. The sigmoid colon measuring 16 cm in length including the perforated area was resected. The diverticulum with rupture was present at the contramesenteric side and was 55 × 35 mm in size (Fig. 1a, b).

**Fig. 2:**
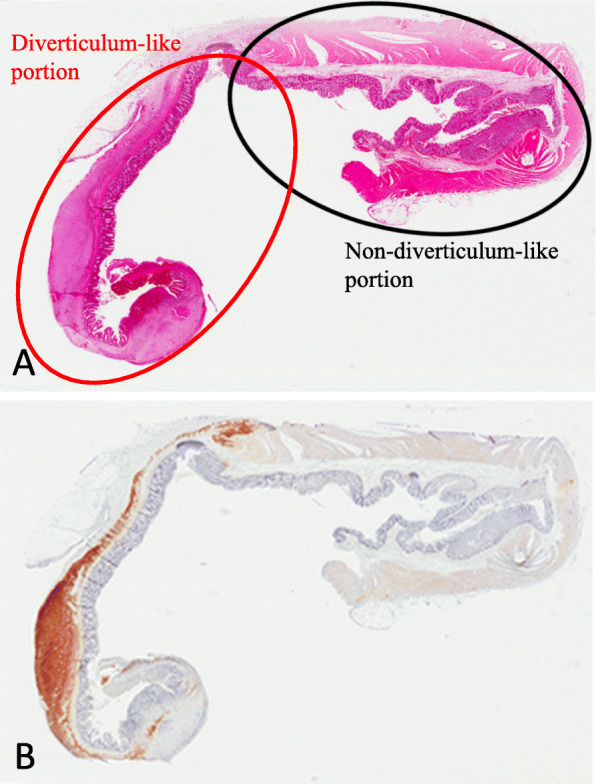
Loupe view of the histological specimens including the diverticular structure with perforation. **a** H&E staining specimen showed the irregular thickness of the wall at the diverticulum-like lesion. **b** KIT immunohistochemistry revealed the flatly proliferating KIT-positive lesion at the diverticulum-like lesion

**Fig. 3:**
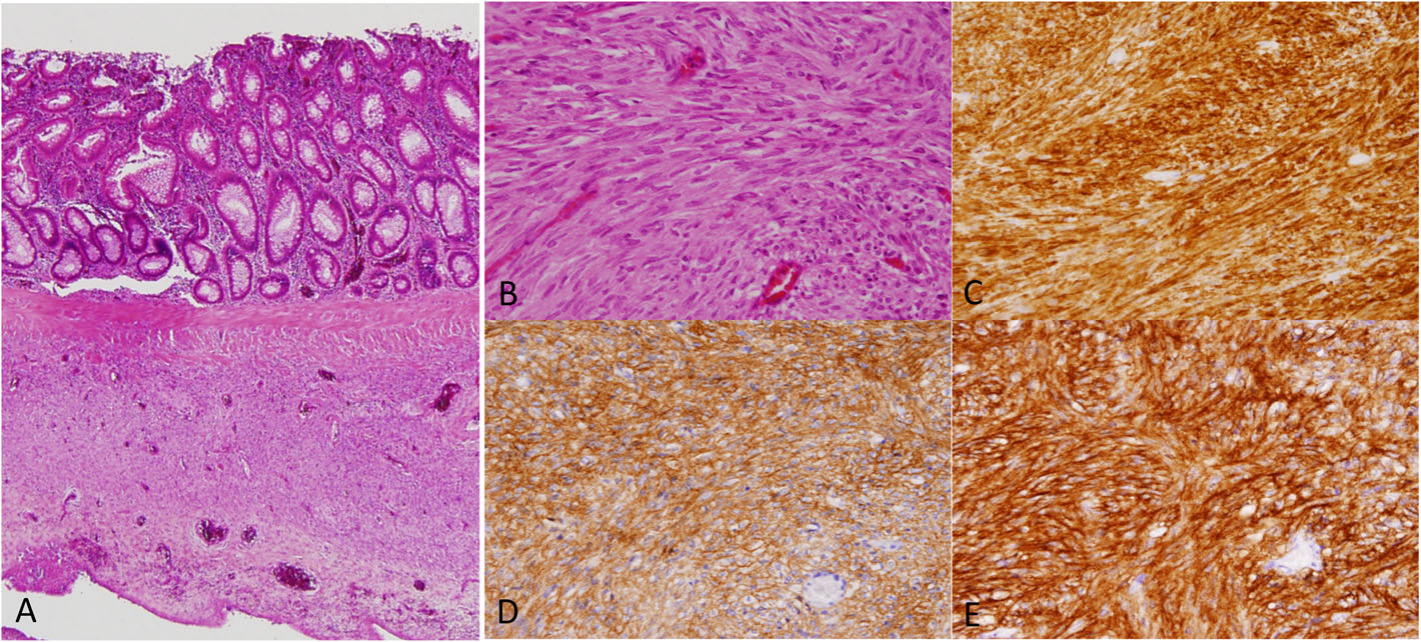
Microscopic examination of the histological specimens of the diverticulum structure. **a** Lower magnification of the H&E staining specimen showed spindle cell proliferation replacing the muscularis propria. **b** Higher magnification of H&E staining demonstrated that the spindle cells were bland and did not have apparent mitoses. **c–e** Immunohistochemistry showed that the proliferating spindle cells were diffusely KIT-positive (**c**), DOG1-positive (**d**), and CD34-positive (**e**)

Microscopically, bland spindle cells were present replacing the whole layer of the muscularis propria without forming an apparent mass at the true diverticulum (Figs. 2a and 3a, b). Necrosis was absent, and mitotic figure was difficult to detect at the lesion (0/50 high-power fields). The non-diverticulum-like portion at the mesenteric side showed normal structure of the colonic wall (Fig. 2a). Basically, the lamina propria, submucosa, and serosa even at the diverticulum-like portion did not show any specific changes (Figs. 2a and 3a), although the whole layer at the perforation site was necrotic. The perforation was observed near the boundary between the normal colonic wall and the lesional wall (Fig. 1a, b).

Immunohistochemical examination revealed that the spindle cells were diffusely positive for KIT (Figs. 2b and 3c), DOG1 (Fig. 3d), and CD34 (Fig. 3e). Alpha-smooth muscle actin was partially positive, but S-100P was negative (data not shown). Since the muscularis propria layer was almost entirely replaced by the KIT-positive spindle cells (Figs. 2b and 3c), desmin was almost completely negative at the lesion. The Ki-67 labeling index was less than 1% (data not shown).

**Fig. 4:**
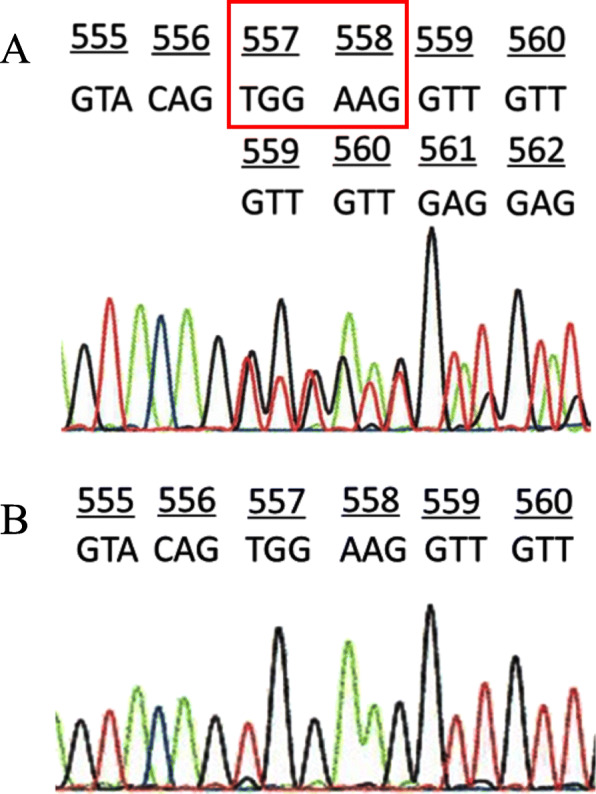
Mutational analysis of the c-*kit* gene using histological specimens. **a** The proliferating spindle cell lesion had a heterozygous deletion of 2 amino acids of codons 557 and 558 at exon 11 of the c-*kit* gene. **b** Surrounding normal mucosal tissue showed a wild-type sequence for the c-*kit* gene

Mutational analysis of the c-*kit* gene at exons 9, 11, 13, and 17 where most mutations are detected in GISTs using macrodissected paraffin-embedded sections showed that the spindle cell lesion had a heterozygous deletion of 2 amino acids at codons 557 and 558 of exon 11 (Fig. 4a). There was no mutation at exons 9, 13, and 17. The surrounding normal mucosal tissue had a wild-type sequence for the c-*kit* gene (Fig. 4b). The PDGFRA mutation was detected neither in the lesion nor in the normal tissue (data not shown).

The patient had mild postoperative paralytic ileus and surgical wound infection but was discharged from the hospital approximately 3 weeks later. He received colonoscopy 6 months after the surgery without special abnormalities and has had no diagnostic imaging thereafter. He has been regularly followed once a month by his family doctor for hypertension and was free from apparent recurrence for 41 months after the surgery.

## Discussion

We experienced an unusual case of planar-type GIST showing flatly distributed and muscularis propria-replacing proliferation at the diverticular structure with perforation in the sigmoid colon.

Most of the sporadic GISTs form nodules of various sizes. On the other hand, patients with germline mutation of the c-*kit* gene show not only multiple GIST nodules in the stomach and small intestine but also flatly proliferating KIT-positive spindle cell lesions at the myenteric plexus layer of the small intestine [8]. The flatly proliferating lesion is called diffuse hyperplasia of ICCs because the lesion was polyclonal in nature [9]. In the present case, we observed a flatly proliferating KIT-positive spindle cell lesion reminiscent of the diffuse hyperplasia of ICCs. However, we could detect the c-*kit* gene mutation in the proliferating spindle cells but not in the normal mucosa, demonstrating that the mutation is a somatic one. Therefore, this is not a case of the diffuse hyperplasia of ICCs observed in the patients with germline mutation of the c-*kit* gene. Flatly proliferating KIT-positive ICC lesions may also be detected at the myenteric plexus layer of the small intestine in neurofibromatosis type 1 (NF1) patients [10], but the present patient did not show any apparent signs of NF1 such as multiple skin neurofibromas and café au lait spots. Moreover, both GISTs and flatly proliferating lesions in NF1 patients do not have c-*kit* gene mutations. Thus, this patient does not seem to be a case of NF1. We concluded that this is a case of sporadic planar-type GIST.

Flatly proliferating ICC lesions in the abovementioned two syndromic settings are usually present as thin layers of spindle cell proliferation along the myenteric plexus occupying the intermuscular layer between the inner circular layer and outer longitudinal muscle layer. In contrast, the entire thickness of the muscularis propria was involved in the flat proliferation of spindle cells in the present case. Thus, the morphological characteristic as well as the genetic abnormality is also different between the present case and the syndromic cases.

Eight similar cases of flatly proliferating and muscularis propria-replacing GISTs including our case have been reported so far as shown in Table 1 [2–7]. Among them, five cases were developed in the sigmoid colon [2–5] and the other three cases were derived from the small intestine [3, 6, 7]. Rectal GISTs account for approximately 10% of all GISTs, but GISTs of the colon are extremely rare with uncertain etiology. Thus, it is interesting but curious that the planar-type GISTs often arise in the colon, especially in the sigmoid colon. The sigmoid colon appears to have a specific environment for planar growth of GISTs.

**Table 1:** Reported cases of flatly distributed and muscularis propria-replacing GISTs

Case	Age/sex	Site	Size (mm)	Diverticulum	Perforation	Mitotic counts (/50HPFs)	Mutation	Recurrence
1 (2)	69/F	S	2–6	+	+	0	NM	−
2 (3)	66/M	S	6	−	−	0	c-*kit* exon 11	−
3 (4)	72/F	S	32	−	+	0	c-*kit* exon 11	−
4 (5)	72/M	S	70	+	+	0	c-*kit* exon 11	−
5 (our case)	68/M	S	23	+	+	0	c-*kit* exon 11	−
6 (3)	59/F	SI	40	+	−	1	c-*kit* exon 11	−
7 (6)	63/F	SI	120	+	−	Less than 1	ND	−
8 (7)	82/M	SI	25	+	+	1	c-*kit* exon 11	−

Numbers in the brackets mean reference numbers

*S* sigmoid colon, *SI* small intestine, *NM* not mentioned, *ND* not detected, *HPFs* high-power fields

The planar-type GIST in this case was present associated with a true diverticular structure. The diverticula are observed in 3 of 5 reported cases of the sigmoid colon and 3 of 3 reported cases of the small intestine. The planar-type GISTs are considered to be closely related to the diverticular structure. Initial lesions of the planar-type GISTs probably occur within the muscularis propria layer like those of usual GISTs. The initial lesion of any types of GISTs might locally alter gut motility and induce disturbed peristalsis. In the case of the sigmoid colon with the narrowest lumen among the various GI sites, the abnormal bowel movement due to the initial lesion might result in stool accumulation and fecaloma formation. The fecaloma might compress the sigmoid colon wall to the outside and locally extend the lumen of the sigmoid colon. Consequently, the dilated diverticular structure might be formed, and it could be finally perforated by the fecaloma compression. The initial lesion of the GIST might be flattened by the oppression of the fecaloma described above, and the oppression itself might stimulate the growth of the initial GIST lesion sideways followed by development of the flatly proliferating GIST. Thus, the sigmoid colon with the narrowest lumen might have the optimal environment for development of the planar-type GISTs.

Kawanowa et al. [11] reported that microscopic small GISTs exist in nearly half of the patients who had undergone total gastrectomy for gastric cancer. The initial lesion of colonic GISTs (i.e., microscopic GISTs) might also develop at any sites of the colon, but the specific condition of the sigmoid colon with the narrowest lumen might induce the proliferation of the initial lesion and the subsequent formation of the unusual planar-type GIST as mentioned above. Therefore, we examined whether the microscopic GISTs were observed in the colon using 50 total colectomy cases resected due to uncontrolled or dysplasia-associated ulcerative colitis with whole sections of 50 to 100 per case. However, we could not detect such an initial lesion of GISTs in every sample. There is a possibility that the patients with ulcerative colitis who had undergone total colectomy are too young to develop the initial lesion of the GISTs. Much more cases with various ages and much more intimate examination might be needed to clarify whether the initial lesions of the planar-type GISTs are present in the colon, especially in the sigmoid colon.

Rectal GISTs are considered to be more aggressive than gastric GISTs. On the other hand, all of the reported cases of planar-type GISTs including this case showed low mitotic figures and low Ki-67 positive rate, and none of the patients had recurrence of GISTs even in the cases with perforation (Table 1) [2, 4, 5, 7]. Thus, the patients with the planar-type GISTs appear to have good prognosis and may not need the adjuvant imatinib therapy. However, the rupture of the usual-type GISTs showing tumor formation often results in peritoneal recurrence of the tumor. We should closely follow the patient to examine the possibility of recurrence. The low proliferative nature of the planar-type GISTs might be associated with the development of them through pressure susceptible growth. Biological differences between rectal GISTs and sigmoid planar-type GISTs have to be clarified.

## Conclusions

We described a case of the planar-type GISTs with a diverticular structure. How the planar-type GISTs develop is not clarified, but we should carefully examine whether the diverticula of the sigmoid colon in the elderly patients have planar-type GISTs.

## Data Availability

All data supporting the findings of this study are available within the article.

## Abbreviations

GIST: Gastrointestinal stromal tumor
GI: Gastrointestinal
HE: Hematoxylin and eosin
ICC: Interstitial cell of Cajal
NF1: Neurofibromatosis type 1
